# Morphology, composition, and mixing state of primary particles from combustion sources — crop residue, wood, and solid waste

**DOI:** 10.1038/s41598-017-05357-2

**Published:** 2017-07-11

**Authors:** Lei Liu, Shaofei Kong, Yinxiao Zhang, Yuanyuan Wang, Liang Xu, Qin Yan, A. P. Lingaswamy, Zongbo Shi, Senlin Lv, Hongya Niu, Longyi Shao, Min Hu, Daizhou Zhang, Jianmin Chen, Xiaoye Zhang, Weijun Li

**Affiliations:** 1 Environment Research Institute, Shandong University, Jinan, 250100 China; 2 School of Environmental Studies, China University of Geosciences, Wuhan, 430074 China; 3 College of Atmospheric Physics, Nanjing University of Information Science and Technology, Nanjing, 210044 China; 4 School of Geography, Earth and Environmental Sciences, University of Birmingham, Birmingham, B15 2TT UK; 5 School of Environmental and Chemical Engineering, Shanghai University, Shanghai, 200444 China; 6 Key Laboratory of Resource Exploration Research of Hebei Province, Hebei University of Engineering, Handan, 056038 China; 7 State Key Laboratory of Coal Resources and Safe Mining, China University of Mining and Technology, Beijing, 100086 China; 8 State Key Joint Laboratory of Environmental Simulation and Pollution Control, College of Environmental Sciences and Engineering, Peking University, Beijing, 100871 China; 9 Faculty of Environmental and Symbiotic Sciences, Prefectural University of Kumamoto, Kumamoto, 862-8502 Japan; 10 Shanghai Key Laboratory of Atmospheric Particle Pollution and Prevention, Department of Environmental Science and Engineering, Fudan University, Shanghai, 200433 China; 11 Key Laboratory of Atmospheric Chemistry of CMA, Institute of Atmospheric Composition, Chinese Academy of Meteorological Sciences, Beijing, 100081 China; 12 Department of Atmospheric Sciences, School of Earth Sciences, Zhejiang University, Hangzhou, 310027 China

## Abstract

Morphology, composition, and mixing state of individual particles emitted from crop residue, wood, and solid waste combustion in a residential stove were analyzed using transmission electron microscopy (TEM). Our study showed that particles from crop residue and apple wood combustion were mainly organic matter (OM) in smoldering phase, whereas soot-OM internally mixed with K in flaming phase. Wild grass combustion in flaming phase released some Cl-rich-OM/soot particles and cardboard combustion released OM and S-rich particles. Interestingly, particles from hardwood (pear wood and bamboo) and softwood (cypress and pine wood) combustion were mainly soot and OM in the flaming phase, respectively. The combustion of foam boxes, rubber tires, and plastic bottles/bags in the flaming phase released large amounts of soot internally mixed with a small amount of OM, whereas the combustion of printed circuit boards and copper-core cables emitted large amounts of OM with Br-rich inclusions. In addition, the printed circuit board combustion released toxic metals containing Pb, Zn, Sn, and Sb. The results are important to document properties of primary particles from combustion sources, which can be used to trace the sources of ambient particles and to know their potential impacts in human health and radiative forcing in the air.

## Introduction

Aerosol particles play an important role in atmospheric chemistry and physics, regional and global climate, and human health^1, 2^. Atmospheric aerosols – both primary and secondary – come from different kinds of anthropogenic and natural sources. Globally, emissions from crop residue, wood, and solid waste combustion are significant sources of primary and secondary aerosol particles. Crop residue and wood combustion release large amounts of gaseous pollutants and carbonaceous aerosol particles including nonmethane volatile organic compounds (NMVOCs), CO, CO_2_, CH_4_, NO_x_, NH_3_, organic carbon (OC), elemental carbon (EC), and metals^3–5^. Combustion of solid waste, especially electronics and plastics, can release large amounts of organic particles (e.g.,﻿ polyaromatic hydrocarbons (PAHs) and brominated phenolic compounds), metals (e.g., Fe, Zn, Mg, Mn, Cu, Ni, Pb, Cr, Hg, and Cd) and various toxic gases (e.g., the toxic dioxins, furans)^6–8^. Many studies have shown that these directly emitted particles from crop residue, wood, and solid waste combustion harm human health and affect climate change by altering direct radiative forcing or acting as cloud condensation nuclei (CCN) to influence indirect radiative forcing^9–12^. Moreover, the oxidation of gaseous precursors further contributes to the formation of secondary particles^13, 14^.

Crop residue and wood as renewable energy are widely used for domestic cooking and heating worldwide. Lanz, *et al*.^15^ reported that residential wood combustion contributed between 17% and 49% to the submicron organic aerosol mass at various rural and urban sites throughout central Europe. In China, the open burning of crop straw occurs frequently after harvesting. It was estimated that crop residues production in China is about 600 Tg every year, approximately 140 Tg of which is burned^16^. Qiu, *et al*.^17^ developed a high-resolution emission inventory of CO (1.03 × 10^4^), CH_4_ (666), NO_x_ (536), NMVOC (1.91 × 10^3^), SO_2_ (87), NH_3_ (138), PM_2.5_ (1.45 × 10^3^), PM_10_ (2.09 × 10^3^), OC (741), BC (137), and CO_2_ (2.45 × 10^5^) from open biomass burning in China, respectively (the values in the parenthesis indicating the emission load of every species, units in Gg per year). Liu, *et al*.^18^ reported that non-fossil sources were responsible for 62 ± 5% and 26 ± 8% of OC and EC by mass in central China, respectively.

The generation of solid waste increases with population growth, economic development and fast urbanization^19^. Christian, *et al*.^20^ estimated that about 2000 Tg/y of garbage are produced globally and about half are incinerated, making this a commonly overlooked major global source of emissions. Because of the lack of waste management services, especially in developing countries, large amounts of garbage are burned along the roadside near residential areas^21^, and the open burning of printed circuit boards occurs frequently in southeastern China for the recovery of metals^22^. Tian, *et al*.^23^ successfully established a multi-year emission inventory of hazardous air pollutants from municipal solid waste incineration in China, showing that large amounts of toxic pollutants (e.g., Sb, Hg, Pb, Cr, Ni, Cd, As, polychlorinated dibenzo-*p*-dioxins and polychlorinated dibenzofurans) were released into the atmosphere.

Although many studies recently have investigated emissions from crop residue, wood and solid waste combustion, they mainly focused on the calculation of emission inventories and chemical composition of particulate matter. To capture the full characterization of aerosol particles, various bulk methods^24, 25^ (e.g., aerosol mass spectrometer (AMS), ion chromatograph (IC)) can be used to acquire mass concentration and composition of particles emitted from different sources; moreover, individual particle methods can further provide detailed information about their morphology and composition^25^. For example, Pagels, *et al*.^26^ applied aerosol time of flight mass spectrometry (ATOFMS) to measure the chemical signatures of individual particles released from solid biofuel combustion. However, limited information is available about the morphology and composition of individual primary particles emitted from these sources, although many studies have well characterized the mixing state, morphology, composition, and aging process of ambient aerosol particles in China^27–31^. We lack sufficient evidence to study the aging process of ambient particles if we do not know the morphology, mixing state and composition of primary particles.

In this study, sixteen different kinds of materials (classified into three groups: crop residue, wood, and solid waste) were burned in a residential stove in our laboratory. The individual primary particles were collected in smoldering and flaming phases and were analyzed with transmission electron microscopy (TEM) coupled with energy-dispersive X-ray spectrometer (EDS). Based on the TEM observations and EDS spectra, we can reveal the morphology, composition, and mixing state of individual primary particles directly emitted from different kinds of materials.

## Results and Discussion

In this study, we classified seven particle types: organic matter (OM), K-rich (KCl, K_2_SO_4_, and KNO_3_), S-rich ((NH_4_)_2_SO_4_), Cl-rich (NH_4_Cl), soot (black carbon), metal (Pb, Zn, Sn, Sb), P-rich, and Br-rich. Details of the classification have been described by Li, *et al*.^25^.

### Individual particles from crop residue combustion

#### Maize straw

**Figure 1:**
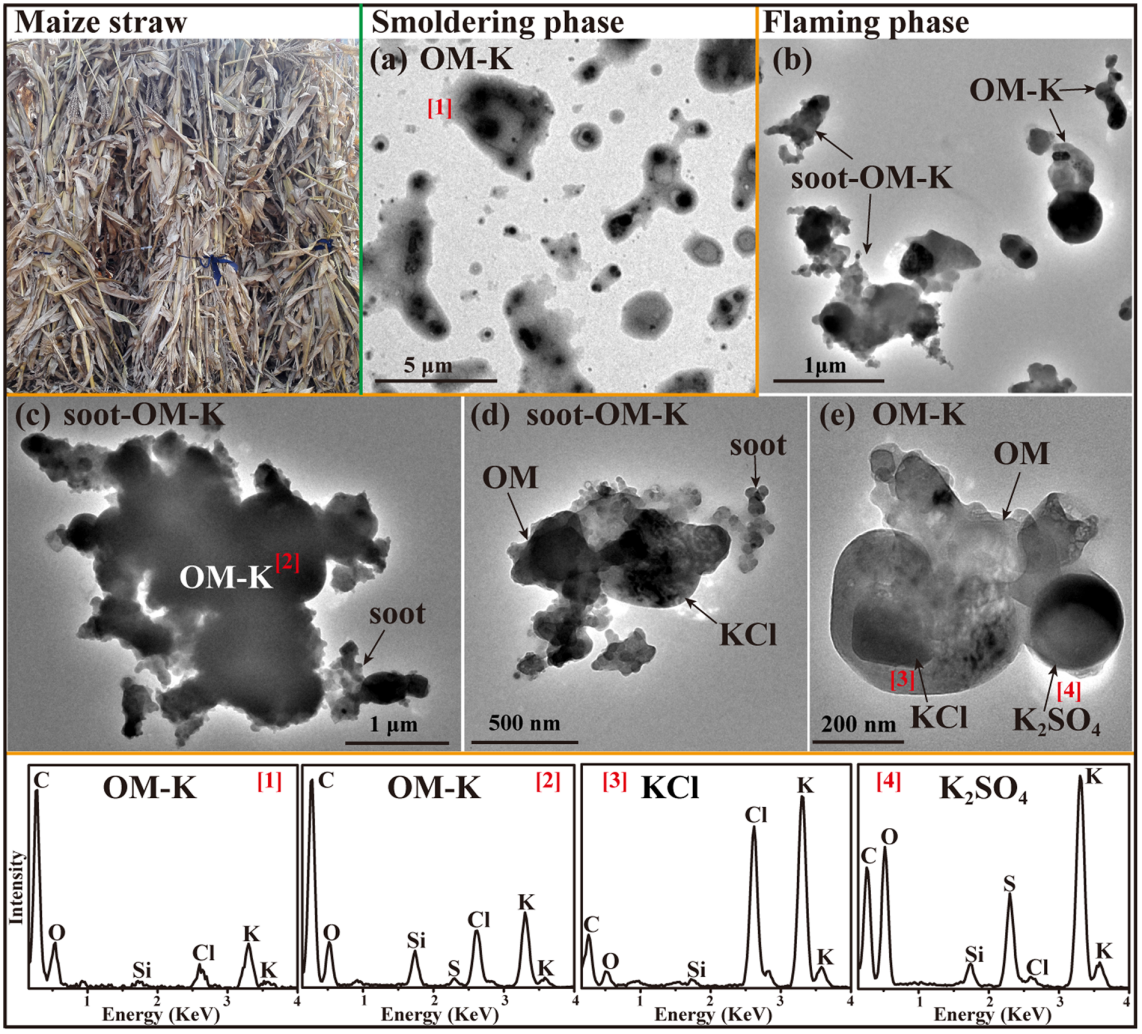
TEM images and EDS spectra of individual primary particles from the combustion of maize straw. (**a**) Low magnification TEM image of gel-like OM particles containing K-rich cores in the smoldering phase; (**b**) Low magnification TEM image of a mixture of OM-K and soot-OM-K particles in the flaming phase; (**c** and **d**) Soot particles internally mixed with OM and KCl in the flaming phase; (**e**) An OM-K particle including KCl and K_2_SO_4_ cores in the flaming phase. The corresponding EDS spectra are indicated by the number in square brackets.

Maize is a widespread Chinese crop, especially in Northeast China and in the North China Plain, and it is harvested between September and November. In rural areas, some maize straw (Fig. 1) is fed to livestock but most is burned in the open agricultural fields or for household cooking and heating^32^. It was estimated that the output of maize straw was 231.66 Tg in all of China in 2004, 22% of which was burned in the fields and 24% burned as domestic fuel^33^. Therefore, emissions from straw burning have to be considered an important source of air pollutants in China. TEM observations show that all the primary particles were a mixture of gel-like OM and K (named as OM-K in Fig. 1a) in the smoldering phase (Table 1). The gel-like OM particles in this study were identified according to the TEM and atomic force microscopy (AFM) analysis (see Supplementary Fig. S1). TEM/EDS clearly show that individual OM-K particles had an OM shell and KCl and/or K_2_SO_4_ cores. In the flaming phase, we found that 53% of primary particles were OM-K internally mixed with soot (named as soot-OM-K) and that the other 47% were OM-K particles (Fig. 1b–e and Table 1). Figure 1c shows a soot particle covered by OM-K, with KCl distributed homogeneously throughout the OM. The soot-OM-K (Fig. 1d) and OM-K (Fig. 1e) particles clearly display crystal KCl and K_2_SO_4_ inclusions, respectively. Obviously, the formation of soot particles stems from the incomplete combustion of straw at these higher temperatures^34^. Based on the EDS spectra, we found that the Si content of primary particles from flaming phase was higher than that from smoldering phase. Besides, we noticed that most soot particles present irregular shapes coated by OM with K-rich inclusions instead of bare chain-like soot aggregation from the diesel engine reported by Neer and Koylu^35^.

**Table 1:** Particle classification, analyzed particle number and percentage from different materials in smoldering/flaming phase.

Material	Combustion phase	Particle type	Particle number	Percentage
Maize straw	smoldering	OM-K	141	100
	flaming	OM-K	57	53
		soot-OM-K	50	47
Wheat straw	smoldering + flaming^*a*^	OM	35	16
		OM-K	124	57
		soot-OM-K	60	27
Cotton straw	smoldering	OM	71	100
	flaming	OM-K	42	70
		soot-OM-K	18	30
Wild grass	smoldering	OM	102	100
	flaming	OM	26	45
		soot-OM	24	41
		Cl-rich-OM/soot	8	14
Common reeds	flaming	OM-K^*b*^	/	/
Apple wood	smoldering	OM	115	100
	flaming	OM-K	70	68
		soot-OM-K	33	32
	burnout^*c*^	soot-OM	29	100
Cypress wood	flaming	soot-OM	177	100
Pine wood	flaming	OM-K	84	100
Pear wood	flaming	soot	66	100
Bamboo	flaming	soot	64	46
		soot-OM-K	75	54
Cardboard	flaming	OM	153	91
		S-rich-OM	15	9
Foam boxes	flaming	soot	106	100
Waste rubber tires	flaming	soot-OM	55	100
Plastic bottles/bags	flaming	soot-OM	105	100
Printed circuit boards	flaming	OM-Br	104	71
		OM-Br-Pb/P/Zn	42	29
Copper-core cables	flaming	OM-Br-soot	83	100

^a^The combustion phases were mixed between smoldering and flaming phases, we present smoldering + flaming here.

^b^The particle number of common reeds cannot be counted because the gel-like OM were connected together.

^c^Burnout refers to the end of the flaming phase.

#### Wheat straw

**Figure 2:**
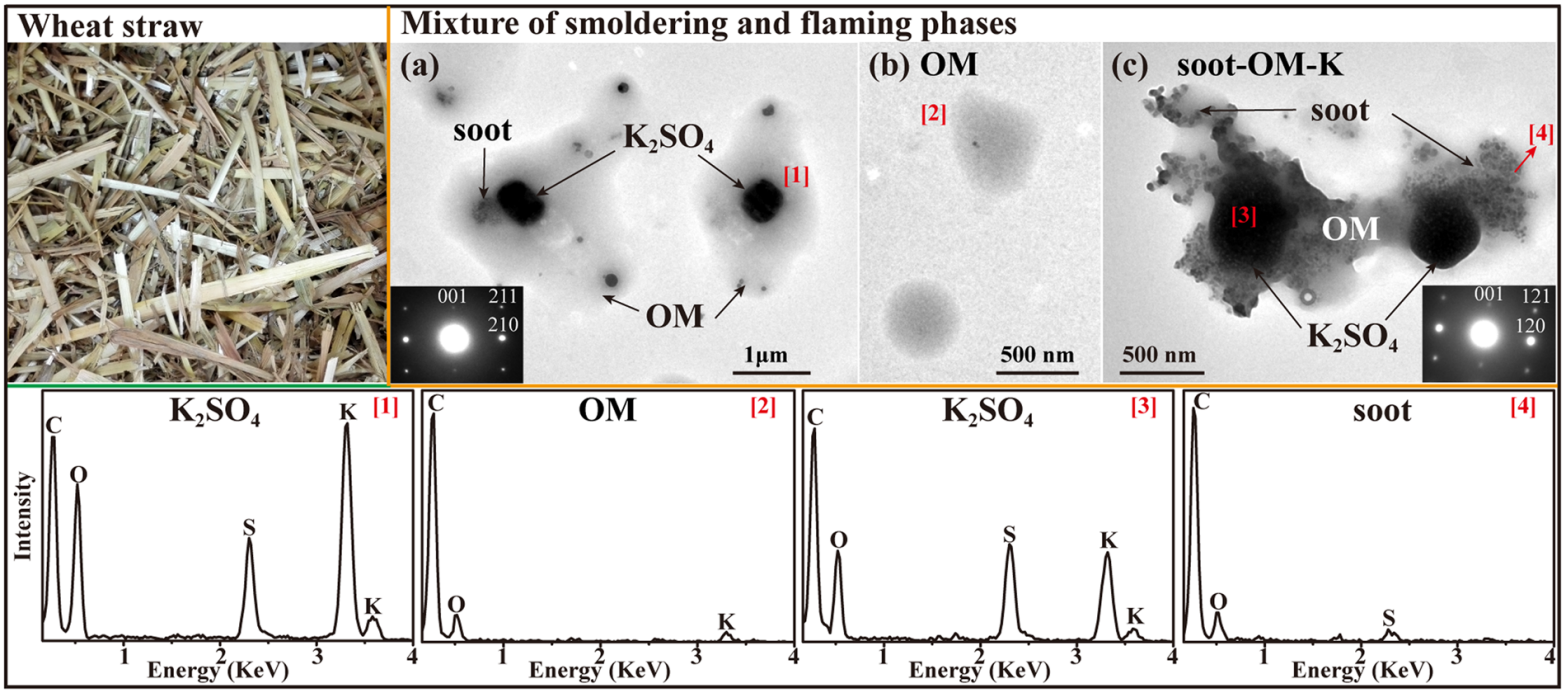
TEM images and EDS spectra of individual primary particles from the combustion of wheat straw. (**a**) Gel-like OM internally mixed with K_2_SO_4_/soot; (**b**) Gel-like OM without any inclusion; (**c**) One irregular particle comprised of soot, K_2_SO_4_, and OM coating. The combustion phase was a mixture of smoldering and flaming phases. The insets show the electron diffraction patterns of K_2_SO_4_ with different K/S ratio. The corresponding EDS spectra are indicated by the number in square brackets.

Wheat is mostly grown in the North China Plain. The amount of wheat residue (Fig. 2) produced in China is about 118.14 Tg each year, about 22% burned in the open fields and 22% burned for cooking and heating^33, 36^. In past decades, farmers commonly burned wheat straw in the agricultural fields, which causes serious air pollution in May–June of each year in eastern China, especially the North China Plain^37, 38^. Because of the rapid combustion of wheat straw, the burning phases were not differentiated and particles were collected in mixed phases between the smoldering and flaming phases. TEM observations and EDS spectra show that about 57% of the primary particles from wheat straw combustion were K_2_SO_4_/KCl cores with gel-like OM shells (Fig. 2a), 16% were gel-like OM particles without inclusions (Fig. 2b), and 27% were a mixture of OM, K_2_SO_4_/KCl, and soot (Fig. 2c and Table 1). The K_2_SO_4_ was confirmed based on the compositions and selected-area electron diffraction patterns (Fig. 2 inset). The morphology of particles shown in Fig. 2a and c were similar to that emitted from maize straw combustion in the smoldering and flaming phases, respectively.

#### Cotton straw

**Figure 3:**
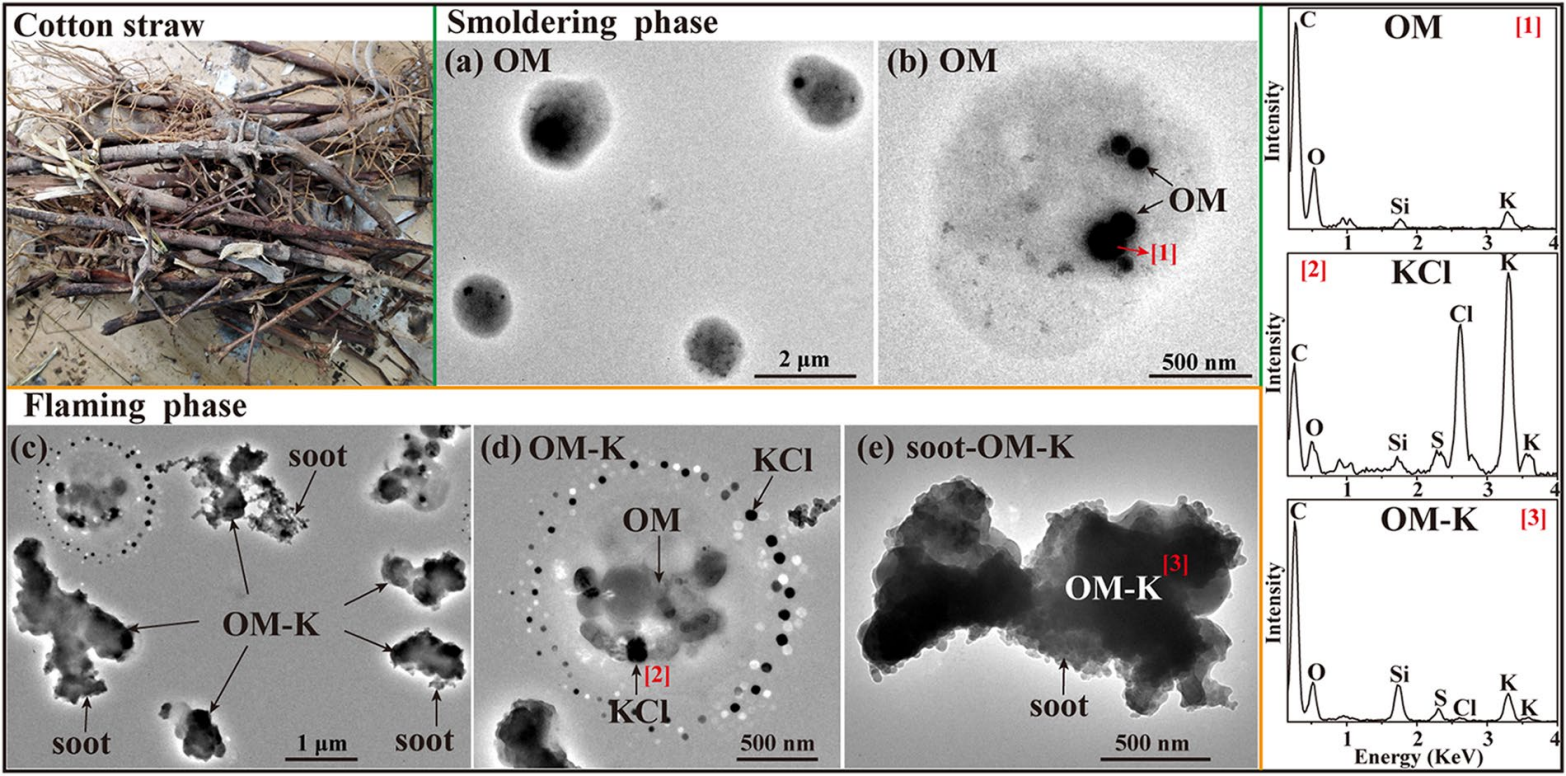
TEM images and EDS spectra of individual primary particles from the combustion of cotton straw. (**a**) Low magnification TEM image of gel-like OM in the smoldering phase; (**b**) A gel-like and transparent OM particle with dark and spherical OM inclusions in the smoldering phase; (**c**) Low magnification TEM image of a mixture of OM-K and soot-OM-K in the flaming phase; (**d**) An OM particle internally mixed with KCl and externally mixed with satellite ring of tiny KCl particles in the flaming phase; (**e**) An OM particle internally mixed with soot in the flaming phase. The corresponding EDS spectra are indicated by the number in square brackets.

The Yangtze River basin, Yellow River basin, and northwestern China are the three main cotton planting regions. The output of cotton straw is about 14.58 Tg annually in China^36^. Because cotton straw (Fig. 3) has a high lignin content and calorific value, it is commonly used as domestic fuel for cooking and heating instead of burning in the open agricultural fields. In the smoldering phase, all the primary particles were OM which looks gel-like and transparent on the substrate (Fig. 3a,b and Table 1). TEM/EDS further shows OM particles with dark and spherical OM inclusions containing C and O with minor Si and K (Fig. 3b). Here we suspect that low-volatile organic compounds (LVOCs) condensing on non-volatile OM particles formed core-shell structures in the smoke plume as it cooled in dispersion. In the flaming phase, 70% of the primary particles were irregular OM-K (Fig. 3c,d and Table 1), and 30% were irregular OM-K internally mixed with soot (Fig. 3c,e and Table 1). Figure 3d clearly shows that the primary OM particles contained KCl inclusions, but the most interesting finding is that the OM-K particles were surrounded by satellite rings of tiny KCl particles. In addition, we did not find external chain-like soot particles but they were more or less mixed with OM-K, as shown in Fig. 3e. We noticed that Si content in the flaming phase was higher than that in the smoldering phase, as similar phenomenon has been mentioned above in the maize straw combustion.

#### Wild grass

**Figure 4:**
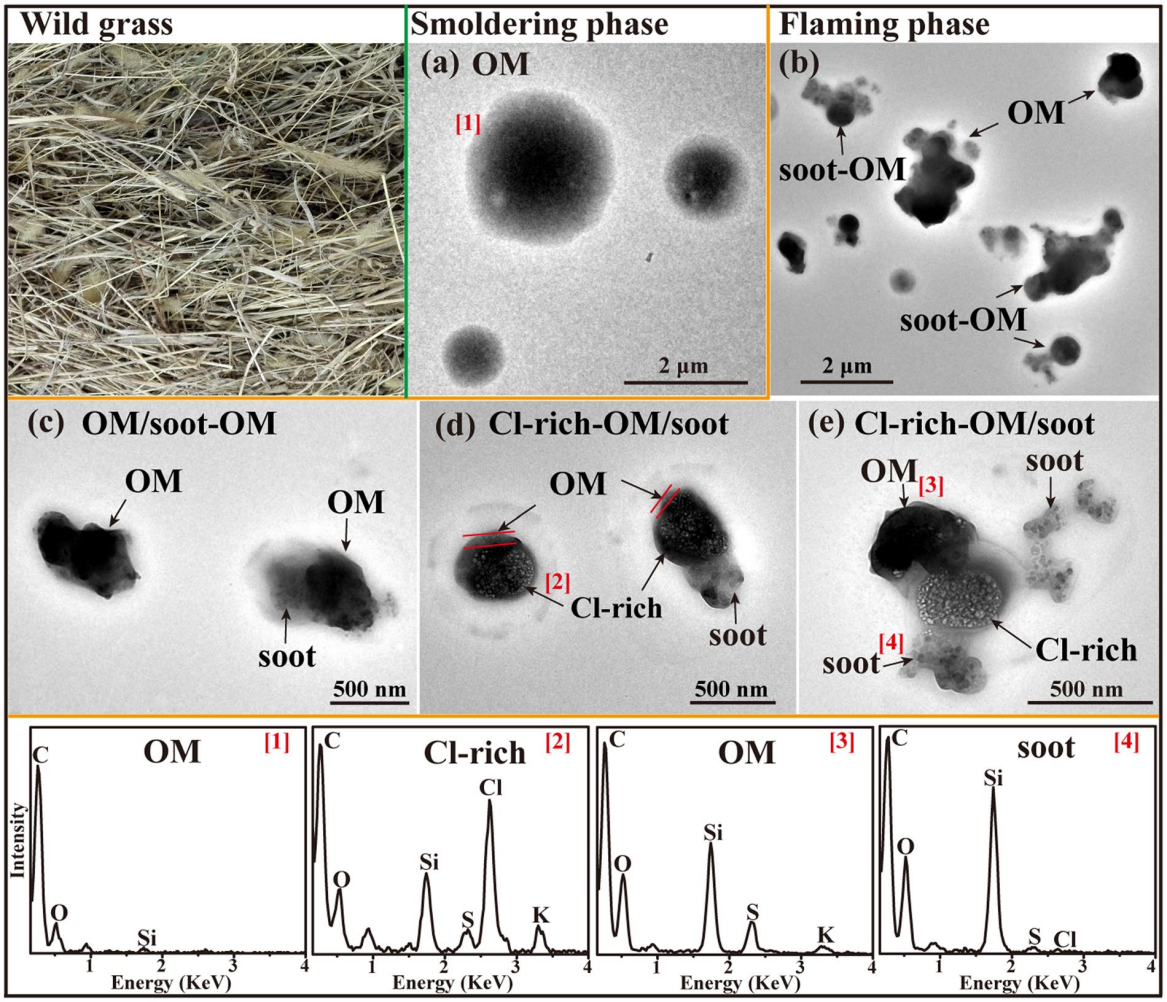
TEM images and EDS spectra of individual primary particles from the combustion of wild grass. (**a**) Low magnification TEM image of the gel-like OM in the smoldering phase; (**b**) Low magnification TEM image of a mixture of OM and soot-OM in the flaming phase; (**c**) Two irrugualr OM and one internally mixed with soot in the flaming phase; (**d**) Two Cl-rich particles with thin OM coating and one internally mixed with soot in the flaming phase; (**e**) An irregular Cl-rich particle internally mixed with OM and soot in the flaming phase. The corresponding EDS spectra are indicated by the number in square brackets.

Grasslands are widely distributed in China, especially in Inner Mongolia. Wild grass (Fig. 4) fires occurring in many grasslands and agricultural fields emit large amounts of air pollutants, especially during the dry winter and spring seasons. The annual amount of grass burned in grasslands in China was estimated to be 52 Tg^39^. In the smoldering phase, TEM observations and EDS spectra show that all the primary particles were gel-like OM, containing high C and minor O and Si without K (Fig. 4a and Table 1). In the flaming phase, 45% of the primary particles were OM, 41% were OM mixed with soot, and 14% were Cl-rich particles (Fig. 4b–e and Table 1). The high resolution TEM images show that Cl-rich particles were beam-sensitive, that they were coated by a thin layer of OM, and that some of them were internally mixed with soot (named as Cl-rich-OM/soot in Fig. 4d,e). EDS shows that the particles from the flaming phase contained rather high amounts of Si, Cl, S, and K; but in the smoldering phase no S, Cl, and K were detected. Interestingly, the Cl content is much greater than K in the Cl-rich particles and the Si content of particles from wild grass burning is considerably higher than that from other biomass emissions produced in the flaming phase. We suspect that the Cl-rich particles could be NH_4_Cl, as similar particles were also reported by Liu, *et al*.^40^ from savanna grass fires in South Africa.

#### Common reeds

**Figure 5:**
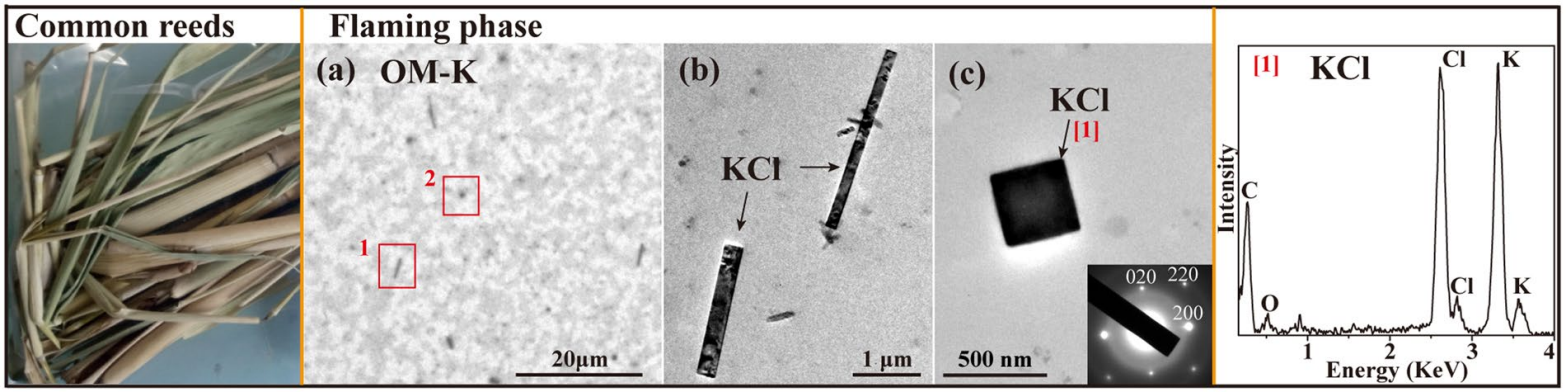
TEM images and EDS spectra of individual primary particles from the combustion of common reeds. (**a**) Low magnification TEM image of gel-like OM particles internally mixed with KCl in the flaming phase; (**b** and **c**) Two rectangle and one square KCl particles corresponding to red square 1 and 2 in (**a**), respectively. The inset shows the electron diffraction patterns of KCl. The corresponding EDS spectra are indicated by the number in square brackets.

Common reeds (Fig. 5) are helophytes widely distributed in rivers, lakes, and wetlands and are commonly used for cooking and heating in some remote areas. In addition, wildfires of common reeds occur occasionally in the natural world. Figure 5 and Table 1 show that square and rectangle shape KCl and gel-like OM were the primary particles in the flaming phase. The KCl was confirmed based on the composition and selected-area electron diffraction pattern (Fig. 5 inset). The high resolution TEM images could not clearly observe the gel-like OM because of their thinness and transparency characteristics (Fig. 5b,c).

### Individual particles from wood combustion

#### Apple wood

**Figure 6:**
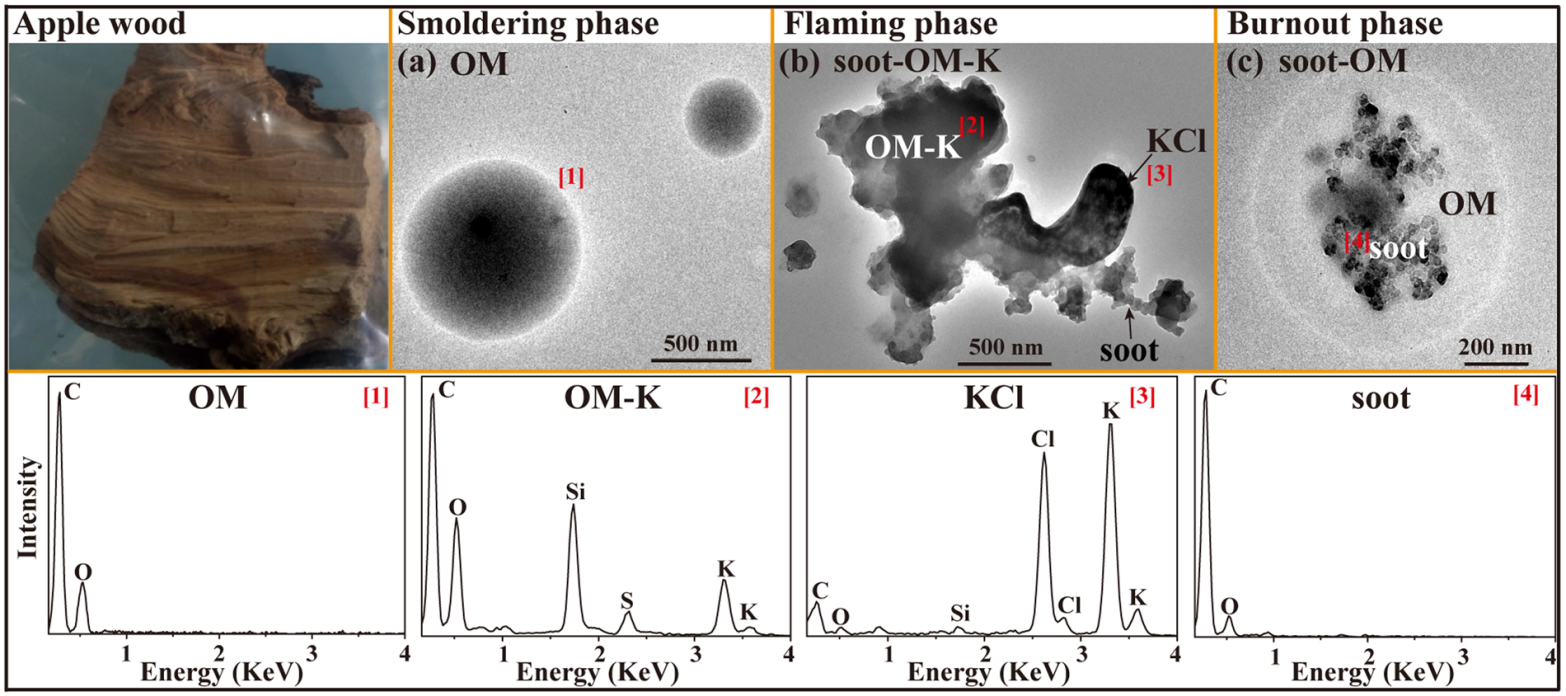
TEM images and EDS spectra of individual primary particles from the combustion of apple wood. (**a**) Two spherical gel-like OM in the smoldering phase; (**b**) An irregular OM particle internally mixed with KCl and soot in the flaming phase; (**c**) A soot particle coated by gel-like and transparent OM in the end of flaming (burnout) phase. The corresponding EDS spectra are indicated by the number in square brackets.

Apple trees are a principal economic crop in hilly areas of China. Apple wood (Fig. 6) is often collected for cooking and heating in winter in rural and mountain areas due to its high calorific value. The total residential consumption of wood in rural China was 182.17 Tg in 2007, according to the data released by National Bureau of Statistics of China (http://www.stats.gov.cn/tjsj/qtsj/hjtjzl/index.htm). In the smoldering phase, TEM observations show that only spherical gel-like OM particles were present (Fig. 6a) and that the corresponding EDS spectra only contained high C with minor O instead of some Si and K in OM particles. These gel-like OM particles shown in Fig. 6a may have come from the condensation of abundant LVOCs emitted in smoldering phase^41^, because LVOCs subjected to high temperatures followed by cooling to room-temperature can condense and produce OM particles^42^. In the flaming phase, the shapes of primary particles changed from spherical to irregular and they contained some Si, S, and K besides C and O. Figure 6b and Table 1 show that 32% of the primary particles were irregular OM internally mixed with KCl and soot (named as OM-K-soot), while the other 68% were irregular OM particles internally mixed with KCl but without soot (named as OM-K). We also noticed that the primary particles collected from the end of flaming phase (named as burnout phase) were cluster-like soot particles with OM coating (named as soot-OM in Fig. 6c). The OM coating looks gel-like and transparent attributing to the condensation of high concentration of LVOCs released from the burnout phase onto soot particles.

#### Cypress and Pine wood

**Figure 7:**
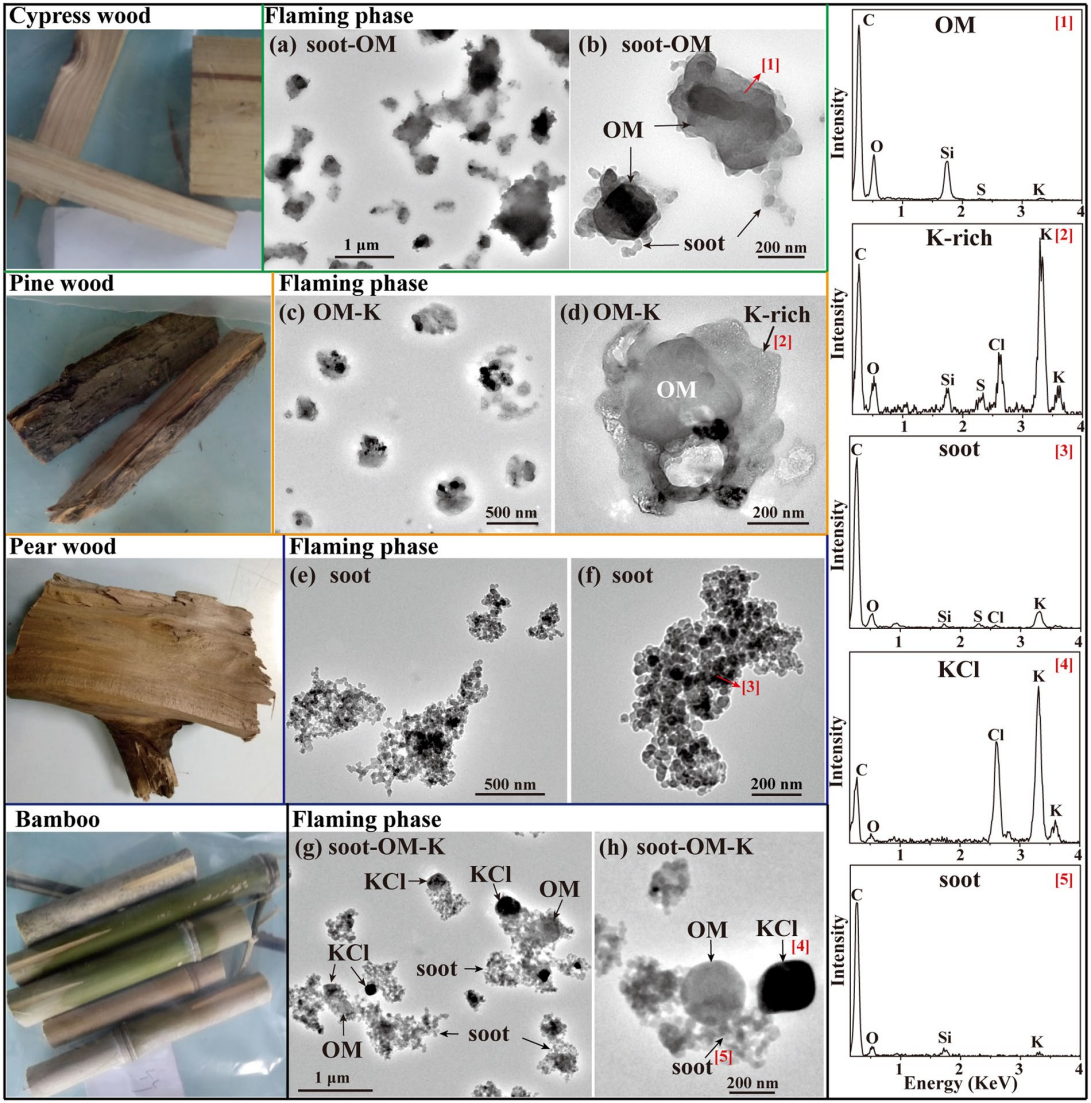
TEM images and EDS spectra of individual primary particles from the combustion of cypress, pine, and pear wood, and bamboo. Low magnification and high resolution TEM images of (**a** and **b**) irregular OM-soot particles from cypress wood, (**c** and **d**) OM-K particles from pine wood, (e and f) bare cluster-like soot particles from pear wood, and (**g** and **h**) cluster-like soot particles internally mixed with KCl and spherical OM from bamboo, in the flaming phase, respectively. The corresponding EDS spectra are indicated by the number in square brackets.

Cypress and pine trees are found mostly in northern China. Cypress and pine wood (Fig. 7) are adequate fuels for cooking and heating for the people living in some rural or mountain areas. The primary particles from cypress wood combustion in the flaming phase were irregular OM-soot particles (Fig. 7a and Table 1). Figure 7b clearly shows that OM was internally mixed with soot particles. EDS spectra show that these particles mainly contained C, O, Si with minor S, Cl, and K. Compared to cypress wood, the primary particles from pine wood combustion in the flaming phase were OM-K particles contained higher S, Cl, and K and soot particles were not observed (Fig. 7c,d and Table 1). The results differed from a previous study which showed that soot was the main component under a well-controlled flaming phase^43^. The reason could be that the pine wood burned very fast and the combustion process in the stove naturally went. Therefore, we speculated that the combustion temperature was not high enough and then led to the lack of soot particles. We noticed that K content of individual particles from pine wood combustion was much higher than Cl and S based on the EDS spectrum. We inferred that additional K may be present in the carbonaceous materials^44^.

#### Pear wood and Bamboo

Pear trees are a main economic crop widely planted in mountainous regions all over China; bamboo is restricted to the warm and humid regions of southern China. Pear wood and bamboo (Fig. 7) are good domestic fuels for cooking and heating. TEM observations show that the morphology and composition of individual particles from pear wood and bamboo combustion were totally different from the types of wood discussed above: the primary particles from apple, cypress and pine wood were mainly OM with soot attached; by contrast, the primary particles from pear wood and bamboo combustion in the flaming phase were cluster-like soot particles which contain lower O and Si than the primary particles from apple, cypress and pine wood. The high resolution TEM images further show that all the particles from pear wood were bare-like soot containing C with minor O, K, Cl, S, and Si (Fig. 7e,f and Table 1). Whereas, 46% of primary particles from bamboo combustion were bare-like soot and 54% of them were soot associated with the crystal KCl and spherical OM particles (Fig. 7g,h and Table 1).

### Individual particles from solid waste combustion

#### Cardboard

**Figure 8:**
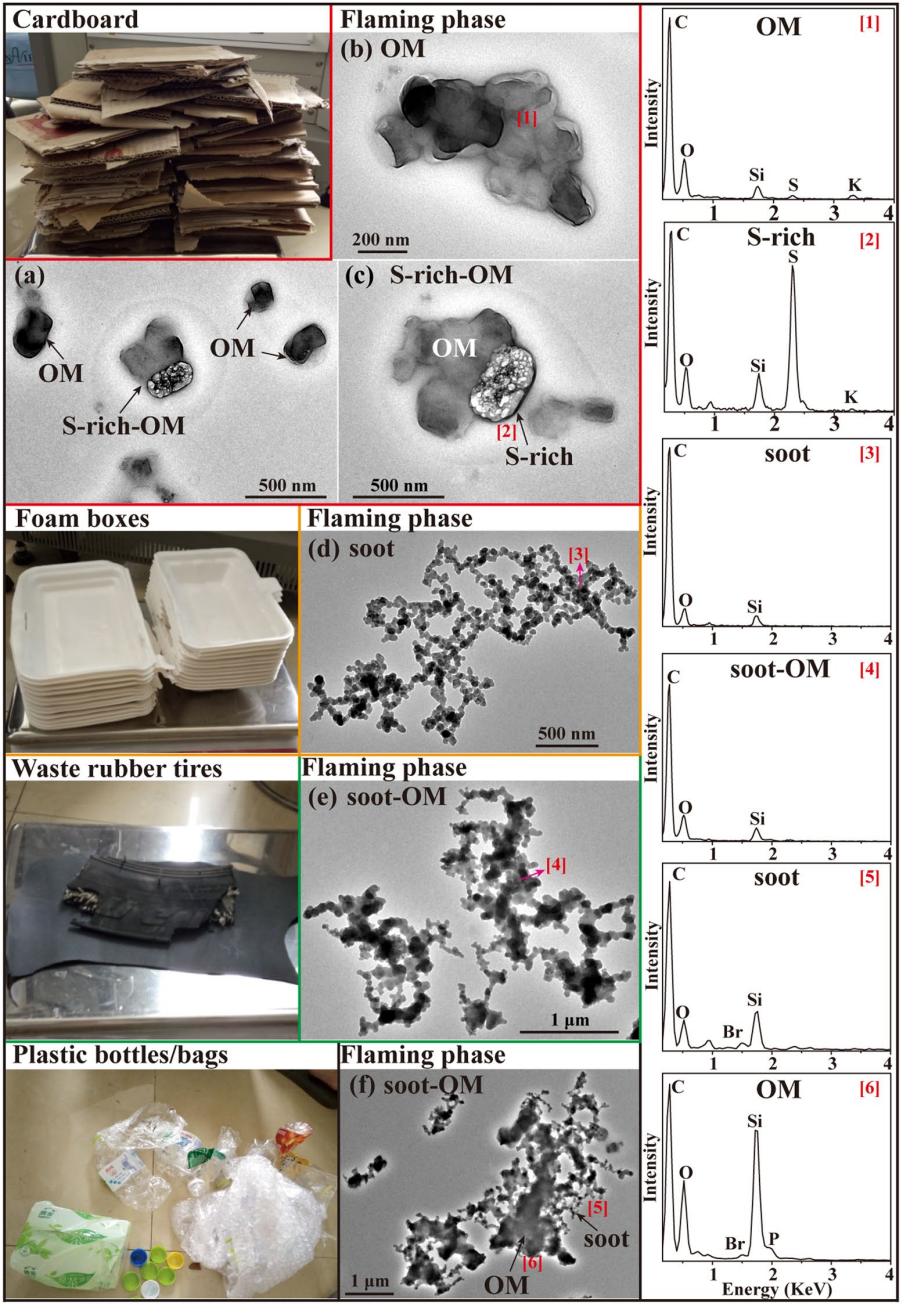
TEM images and EDS spectra of individual primary particles from the combustion of cardboard, foam boxes, waste rubber tires, and plastic bottles/bags. (**a**) Low magnification TEM image of OM and S-rich-OM from cardboard in the flaming phase; (**b**) An irregular OM particle from cardboard in the flaming phase; (**c**) An S-rich particle internally mixed with OM from cardboard in the flaming phase; (**d**) A bare chain-like soot particle from foam boxes in the flaming phase; (**e**) Soot particles coated by thin OM from waste rubber tires in the flaming phase; (**f**) A soot particle coated by thick OM from plastic bottles/bags in the flaming phase. The corresponding EDS spectra are indicated by the number in square brackets.

Cardboard (Fig. 8) is widely used for the outer packing of containers and boxes. The main components of cardboard are C, O, H, S, Al, alkali, and alkaline earth metals^45^. In the flaming phase, we found that 91% of the primary particles from cardboard combustion were irregular OM particles which contain C, O with minor Si, S, and K (Fig. 8a,b and Table 1). The interesting finding is that 9% of the primary particles were S-rich internally mixed with OM (named as S-rich-OM in Fig. 8a,c), whereas ambient S-rich particles were considered as secondary particles from conversion of gaseous SO_2_ in the atmosphere^46^. Figure 8a–c show that these dry OM particles displayed distinct rims on the substrate, implying that they retained soluble OM on particle surfaces.

#### Foam boxes, waste rubber tires, and plastic bottles/bags

Foam boxes and plastic bottles/bags (Fig. 8) are consumed in our daily lives; waste rubber tires (Fig. 8) have proliferated recently with the increasing number of vehicles. All these materials should either be recycled or burned in garbage incineration plants, but many of them are burned at the garbage sites or in the domestic stoves. Based on our investigations, foam boxes are made of polystyrene, plastic bottles/bags are made of polyethylene or polyethylene terephthalate, and waste rubber tires comprise natural rubber and some chemical additives (e.g., carbon black, aromatic amine antioxidants). The primary particles from foam boxes combustion in the flaming phase were bare chain-like soot particles containing C with minor O and Si (Fig. 8d and Table 1). Figure 8e shows that the soot particles from waste rubber tires combustion were more compact than that from foam boxes combustion. The boundaries between primary spherules in the soot aggregates were unclear because soot particles were coated by OM containing C and minor O and Si. Compared to the two kinds of materials above, TEM/EDS data show soot particles from plastic bottles/bags combustion were internally mixed with much more OM and contained a higher content of O and Si (Fig. 8f). It should be noted that the EDS spectra of particles from plastic bottles/bags combustion displayed a low peak of Br, which might be attributed to the low content of brominated flame retardants (BFR).

#### Printed circuit boards

**Figure 9:**
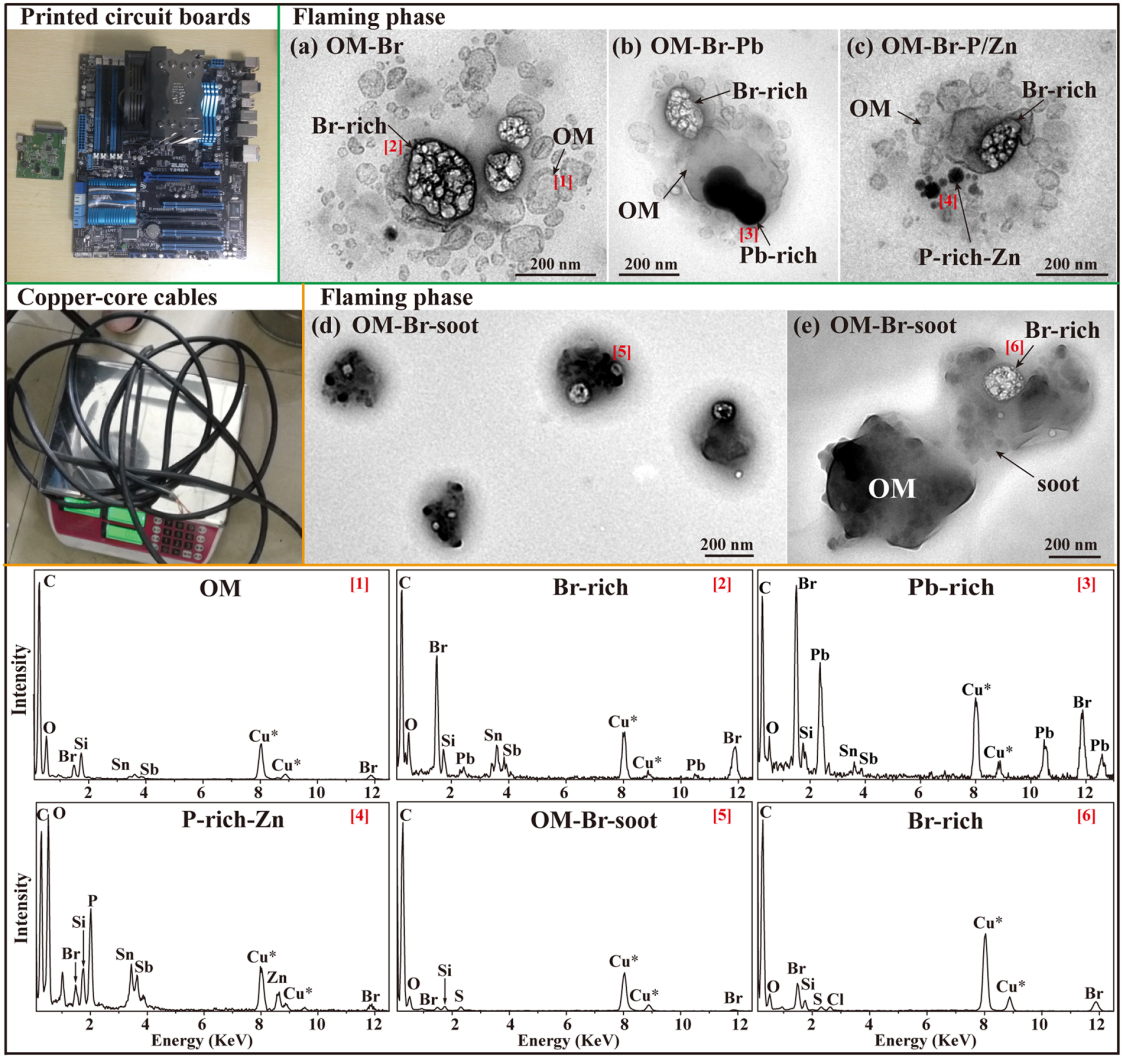
TEM images and EDS spectra of individual primary particles from the combustion of printed circuit boards and copper-core cables. (**a**) Br-rich particles internally mixed within bubble-like OM (Sn, Sb) from printed circuit boards in the flaming phase; (**b** and **c**) Bubble-like OM (Sn, Sb) internally mixed with Br-rich, Pb-rich, P-rich and Zn-rich from printed circuit boards in the flaming phase; (**d** and **e**) Low magnification and high resoluton TEM images of OM-Br-soot particles from copper-core cables in the flaming phase. The corresponding EDS spectra are indicated by the number in square brackets. The Cu* peaks in the EDS spectra are not analyzed because of the interference from the copper TEM grids.

Printed circuit boards (Fig. 9) are essential to all modern electronic products, such as computers, television sets, cell phones etc. A printed circuit board is normally composed of 40% metals (e.g., Cu, Al, Pb, Ni, Zn, Sb, Sn, and Au), 30% polymers (e.g., polyethylene, polypropylene, and polyester), and 30% ceramic^47^. The estimated production of electronic waste is about 40 million tons per year globally, 3% of which by weight is printed circuit boards^48^. Most global electronic waste is recycled in the developing countries, especially southern China and India. The high-temperature combustion method is widely used to dispose printed circuit boards due to its high recycling ratio of precious metals and low cost^48^. This key disposal process can release large amounts of air pollutants and hazardous substances which affect the air quality and cause serious health effects on workers and nearby residents. In the flaming phase, we found that 71% of primary particles from printed circuit board combustion were a mixture of OM and Br-rich, which contained high C and Br with minor O, Si, Sn, and Sb (Fig. 9a and Table 1). In addition, 29% of particles were internally mixed with some additional particles, such as Pb-rich, P-rich, and Zn-rich (Fig. 9b,c and Table 1). TEM observations show that Br-rich particles were coated with OM and sensitive to the strong electron beam and that bubble-like OM were dispersed around Br-rich particles (Fig. 9a–c). EDS further shows that these OM particles homogeneously contained Sn and Sb, which could form some OM-metal complexes. To our knowledge, this mixture of the OM (Sn, Sb) and Br-rich particles and their morphology have not been previously reported. The Br-rich particles may have been generated from the combustion of BFR, which is added into the flammable plastic material to increase the fire resistance^49^. The components of these particles acquired by the TEM/EDS in this study are consistent with the results reported by Bi, *et al*.^11^.

#### Copper-core cables

Copper-core cables (Fig. 9) are widely used in electrical power transmission and telecommunications. The recycling procedure of copper-core cables normally recovers the copper core but burns the black cable sheaths made of a special rubber containing chlorosulfonated polyethylene. TEM observations show that OM internally mixed with Br-rich and soot formed the cores which were covered by a transparent OM coating in the flaming phase (Fig. 9d,e). EDS shows that OM mainly contained C with minor O, Si, Br, S and Cl. The copper-core cables contain BFR to reduce the flammability, therefore, particles from copper-core cable combustion have similar Br-rich inclusions as described in the above printed circuit boards section.

## Comparison of aerosol particles from different materials

**Figure 10:**
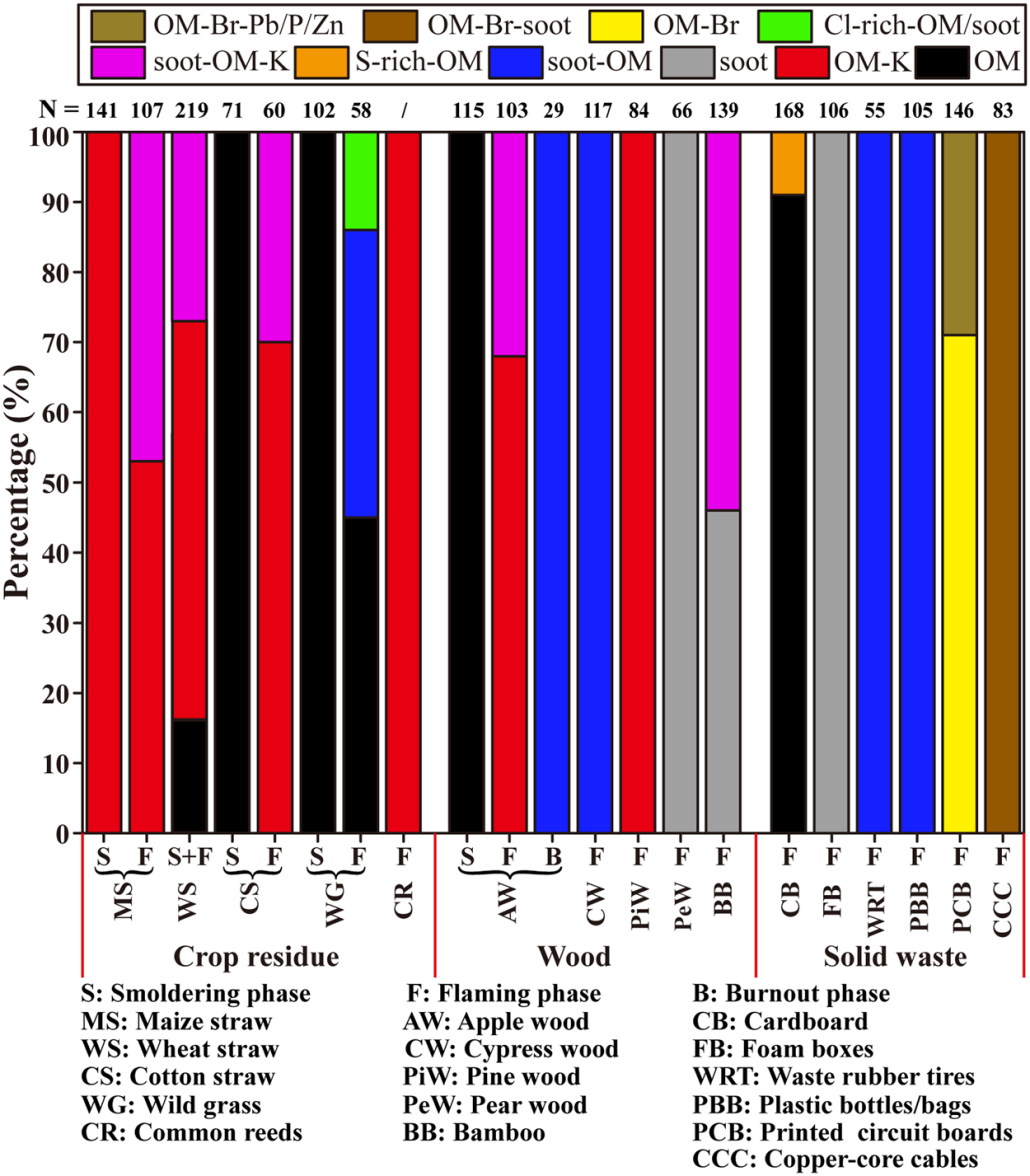
Percentages of typical primary particles emitted from different materials’ combustion with the smoldering and flaming phases. The number of the analyzed particles is shown above each column. The particle number of common reeds cannot be counted because the gel-like OM particles were in contact with each other.

Figure 10 and Table 1 show the obvious differences between the smoldering and flaming phases of the same material and among crop residue, wood, and solid waste in the same combustion phase. In smoldering phase, we found that primary particles from cotton straw, wild grass, and apple wood combustion were all OM with low or without K content. Particles from maize straw combustion in the smoldering phase were OM-K particles, which contained K-rich (major KCl and minor K_2_SO_4_) inclusions. Considering the low combustion temperature in the smoldering phase, we speculate that the OM were generated mainly from the condensation of gaseous organics released by the fuel pyrolysis. Contrariwise, in the flaming phase, primary particles from maize straw, cotton straw, apple wood, and common reeds combustion mainly consisted of irregular soot-OM and soot-OM-K. It should be noted that wild grasses did not exhibit K-rich particles but Cl-rich-OM/soot particles instead. In this study, the primary particles from wheat straw combustion were OM, OM-K, and soot-OM-K in the mixed phases between the smoldering and flaming phases.

Soot formation is linked to the formation of PAHs and to the development of the flame during combustion^50, 51^. Therefore, in the flaming phase, organic matter burns in the diffusion flame to produce PAHs and then forms soot particles instead of condensing^42^. We found abundant K-rich particles in some materials in the flaming phase, in good agreement with Pagels, *et al*.^26^, who observed higher K_2_Cl^+^ peaks of particles emitted from the biofuel combustion in the flaming phase than in the smoldering phase. K-rich particles were produced through the condensation of ash elements (e.g., K, Cl and S) which volatilized in combustion due to the elevated temperatures^52^. It should be noted that the O/C ratio of primary OM particles from crop residue burning is much lower than the ambient particles collected in urban air^37^. In addition, we found that Si was a common component in the primary OM particles from biomass burning and the Si content was higher in the flaming phase than in the smoldering phase. Si was also frequently detected in the field observations and the samples collected from biomass burning in the laboratory reported in the previous literatures^31^. The reason is that Si as a beneficial nutrient for plant growth is taken up by plants via the transpiration stream in the form of silicic acid, and therefore high content of Si is deposited in plant body as solid, amorphous and hydrated silica (SiO_2_·*n*H_2_O)^53^. A previous research showed that SiO_2_ was the most abundant composition in the ash of biomass burning^54^. We speculate that only a small amount of SiO_2_ was volatilized in combustion process and homogeneously mixed within OM particles because of the high melting temperature. Moreover, we noticed that grasses combustion emitted a high content of Si in OM (Fig. 4) because grasses have an enhanced uptake of Si to defense herbivore damage^55^.

Rowell^56^ divided woods into two groups: softwoods mainly from conifers and hardwoods from flowering plants. In this study, cypress and pine woods were classified as softwoods, and pear wood and bamboo were hardwoods. By comparing the primary particles from wood combustion in the flaming phase, we found that particles from pear wood and bamboo (hardwoods) were cluster-like soot with certain amounts of OM, whereas particles from cypress and pine wood (softwoods) mainly contained OM with certain amounts of soot. Shen, *et al*.^57^ measured the emission factors of OC and EC from residential wood combustion and showed that EC/OC ratios for bamboo, cypress, and pine were about 9.2, 0.86 and 1.57, respectively. These results are generally consistent with the TEM observations.

Compared to crop residue and wood combustion, the particles from solid waste combustion in the flaming phase displayed totally different morphologies and compositions. Particles from cardboard were OM with small amounts of S-rich-OM/soot. For foam boxes, waste rubber tires, and plastic bottles/bags, chain-like soot was the dominant particle type. We also noticed that foam boxes emitted bare-like soot, whereas waste rubber tires and plastic bottles/bags emitted soot internally mixed with slight OM. The particles from printed circuit board and copper-core cable combustion were dominated by OM-Br. Interestingly, individual particles from copper-core cables frequently had soot inclusions and those from printed circuit boards did not contain soot but certain amounts of Pb, P, and Zn. Among the plastics, the combustion of polystyrene can release higher amounts of PAHs than the other plastics because of its aromatic structure^58, 59^. This is a plausible explanation why foam boxes made of polystyrene released abundant pure soot particles. Soot particles were not observed in printed circuit board samples, which may be attributed to the low combustion temperatures from the higher content of BFR.

### Conclusions and Atmospheric Implications

Crop straws and woods are widely used for cooking and heating in rural regions of China. The crop residues after harvesting are commonly burned in open agricultural fields, and the solid waste is often burned along the roadsides or at the garbage sites. Crop residue, wood and solid waste combustion have caused wide concerns because of their negative influences on climate and human health. Crop residue and wood combustion are estimated to be one of the largest sources for emissions of OC and EC in all of China. Solid waste combustion releases large amounts of PAHs, dioxins and heavy metals. This particulate matter affects the radiative forcing on regional and global scales^60^ and causes severe haze episodes at the regional scale^37^ and various effects on human health^61^.

In this study, we found the individual primary particles emitted from different materials were complicated, even in different combustion phases. We further summarized some rules based on our study: (1) Particles from crop residues and apple wood combustion are mainly OM in the smoldering phase but are a mixture of soot, OM, and K-rich in the flaming phase. (2) Wild grass combustion releases certain amounts of Cl-rich-OM/soot particles. (3) The main particles from combustion of softwoods and hardwoods in the flaming phase are OM particles attached with certain soot and soot particles thinly coated by OM, respectively. (4) Cardboard combustion produces a mixture of OM and certain S-rich particles. (5) Some household waste such as foam boxes, waste rubber tires, and plastic bottles/bags are highly flammable materials and release large amounts of chain-like soot thinly coated by OM. (6) The combustion of BFR-containing materials (e.g., printed circuit boards and coper-core cables) mainly release OM particles with Br-rich inclusions.

The results in this study will be important to improve our knowledge of the properties of individual primary particles from the combustion of different sources in the smoldering and flaming phases, which is the basis to further understand the aging process and sources of ambient aerosols in some polluted areas. This study shows that the flammable materials can emit large amounts of soot and internally mixed OM particles. To date, there is a hot debate on the role of soot particles in climate because of the lack knowledge about the shape variations and mixing states of soot particles^62, 63^. According to the TEM analysis, our study clearly revealed that many freshly emitted soot particles more or less associated with OM from different sources instead of the completely bare-like soot particles. In the future work, we can characterize the mixing state and fractal dimension of soot particles using statistical scaling law^9, 64^. These data can be used to correct the parameters in the current climate modelling, which are important to estimate the radiative forcing and improve our understanding of the effects of soot on climate change. Moreover, our study provided the details of physicochemical properties of fresh particles, which is helpful to understand how emissions of different materials influence indoor air quality.

## Materials and Methods

### Materials

Sixteen kinds of materials were classified into three different groups: crop residues (e.g., maize straw, wheat straw, cotton straw, common reeds, and a mixture of various wild grass) collected from rural areas in Shandong and Hebei province; four types of wood (e.g., apple wood, cypress wood, pine wood, and pear wood) collected from rural areas in Shandong and Hebei province, and bamboo collected from rural areas in Jiangsu province; and solid wastes (e.g., cardboard, plastic bottles, foam boxes, waste rubber tires, printed circuit boards, and copper-core cables) collected from three garbage sites in urban area. All the materials were dried in a ventilated place and cut into small pieces.

### Combustion experiments

The combustion facility built in the laboratory consists of four main parts: residential stove, dilution tunnel, sampling chamber, and individual particle sampler (see Supplementary Fig. S2). In the combustion experiments, the combustion process can be divided into smoldering and flaming phases: smoldering phase, in the beginning after ignition, has low combustion temperature releasing thick smoke without obvious flame; flaming phase has an obvious flame and high temperature after smoldering phase. We noticed that some other studies defined the flaming phase followed by the smoldering phase for biomass burning^65, 66^. It should be noted that a small-scale burning was conducted in the residential stove in this study and the combustion conditions may differ from the wild biomass burning. Maize straw, wheat straw, cotton straw, wild grass and apple wood generally exhibited both smoldering and flaming phases, both of which were sampled. In contrast, common reeds, cypress wood, pine wood, pear wood, bamboo, cardboard, plastic bottles, foam boxes, waste rubber tires, printed circuit boards, and copper-core cables only displayed a flaming phase due to their rapid combustion. These combustion experiments were repeated twice for each type of material.

### Individual particle collection

After ignition in the residential stove, different materials were burning under the actual situation without any control about the combustion temperature and oxygen supply. Part of the smoke released from the stove entered the sampling chamber through the dilution tunnel when the pump connected to the sampling chamber was switched on. The smoke was diluted 10~25 times with the dry and clean air and stayed about 30~40 s in the dilution tunnel and sampling chamber until the temperature of the diluted smoke was 3~5 °C higher than the room temperature before we collected samples. Individual primary particles were collected on copper TEM grids covered by carbon film (carbon type-B, 300-mesh copper, Tianld Co., China) using a single-stage cascade impactor (jet nozzle diameter 0.3 mm, air flow rate 1.0 L·min^−1^) connected to the sampling chamber through a pipe. The individual particle sampler has a 50% collection efficiency for 80 nm of aerodynamic diameter if the particle density is 2 g·cm^−3^. To collect the fine particles, we installed one pre-filter with polycarbonate membrane (Whatman, UK) with 2 μm pores before the particles were pumped into the single particle impactor. Therefore, this system only collected the fine particles (<2 μm) in the fresh smoke plume. After collections, the samples were placed in a sealed, dry plastic tube and stored in a desiccator at room temperature. The blower was used to flush the combustion facility after each combustion test.

### Individual particle analysis

Individual particle samples were analyzed by a transmission electron microscope (TEM) at 200 kV accelerating voltage (JEM-2100, JEOL Ltd., Japan) equipped with an energy-dispersive X-ray spectrometer (EDS). The distribution of aerosol particles on TEM grids was not uniform, with coarse particles near the center of the grid and with fine particles on the periphery. Therefore, to ensure that the analyzed particles were representative, three to four areas were chosen from the center and periphery of the sampling spot on each grid. TEM images were taken to observe the morphology and mixing state of individual primary particles. EDS was used to semi-quantitatively determine elemental composition of particles. Elements heavier than carbon can be detected, but the Cu peak in the EDS spectra was not analyzed because of the interference of the copper TEM grids substrate. For some particles, EDS data were combined with selected-area electron diffraction (SAED) to verify their identities. Moreover, atomic force microscope (AFM, Digital Instrument Co., Ltd., USA) was used to measure thickness of individual typical particles. The method well confirmed gel-like and irregular particles based on their thickness on the substrate (see Supplementary online).

### Data availability

All the data presented in this paper are available upon request. Please contact the corresponding author (liweijun_atmos@gmail.com or liweijun@zju.edu.cn).

## Electronic supplementary material

Supplementary Information
