# Supplementary material for: Morphology, composition, and mixing state of primary particles from combustion sources — crop residue, wood, and solid waste
Source: Sci Rep. 2017 Jul 11;7:5047. doi: 10.1038/s41598-017-05357-2 (PMC5505958; doi:10.1038/s41598-017-05357-2)
Supplement: Supplementary file 1 — Supplementary Information [file 41598_2017_5357_MOESM1_ESM.pdf]

Supplementary Information of

Morphology, composition, and mixing state of primary particles from combustion sources — crop residue, wood, and solid waste

Lei Liu[1], Shaofei Kong[2,3], Yinxiao Zhang[1], Yuanyuan Wang[1], Liang Xu[1], Qin Yan[3], A.P. Lingaswamy[1], Zongbo Shi[4], Senlin Lv[5], Hongya Niu[6], Longyi Shao[7], Min Hu[8], Daizhou Zhang[9], Jianmin Chen[10], Xiaoye Zhang[11], and Weijun Li[1,12,*]

[1]Environment Research Institute, Shandong University, Jinan, 250100, China

[2]School of Environmental Studies, China University of Geosciences, Wuhan, 430074, China

[3] College of Atmospheric Physics, Nanjing University of Information Science and Technology, Nanjing, 210044, China

[4]School of Geography, Earth and Environmental Sciences, University of Birmingham, Birmingham, B15 2TT, UK

[5]School of Environmental and Chemical Engineering, Shanghai University, Shanghai, 200444, China

[6] Key Laboratory of Resource Exploration Research of Hebei Province, Hebei University of Engineering, Handan, 056038, China

[7] State Key Laboratory of Coal Resources and Safe Mining, China University of Mining and Technology, Beijing, 100086, China

[8] State Key Joint Laboratory of Environmental Simulation and Pollution Control, College of Environmental Sciences and Engineering, Peking University, Beijing, 100871, China

[9] Faculty of Environmental and Symbiotic Sciences, Prefectural University of Kumamoto, Kumamoto, 862-8502, Japan

[10]Shanghai Key Laboratory of Atmospheric Particle Pollution and Prevention, Department of Environmental Science and Engineering, Fudan University, Shanghai, 200433, China

[11]Key Laboratory of Atmospheric Chemistry of CMA, Institute of Atmospheric Composition, Chinese Academy of Meteorological Sciences, Beijing, 100081, China

[12]Department of Atmospheric Sciences, School of Earth Sciences, Zhejiang University, Hangzhou, 310027, China

*Corresponding Email: liweijun@zju.edu.cn (W. Li)

AFM analysis

Atomic force microscopy (AFM) with a tapping mode was adopted to analyze aerosol particles under ambient conditions. AFM, a Multimode Nanoscope-IIIa Scanning Probe Microscope (Digital Instrument Co., Ltd., U.S.A.), can detect the three-dimensional morphology of particles. The AFM settings contain imaging forces between 1 and 1.5 nN, scanning rates between 0.5 and 0.8 Hz, and scanning range sizes at 10 μm with a resolution of 512 pixels per length. After the AFM analysis, morphology and composition of the same particles was confirmed by TEM. The NanoScope Analysis software can automatically obtain bearing area (*A*), bearing volume (*V*) and bearing height (*H*) of each analyzed particle. The 3D AFM image and the corresponding TEM image are shown in Fig. S1 to illustrate the identification of the irregular and gel-like OM. The gel-like OM has a low viscosity because it is hydrated[1]. Therefore, when deposited onto the copper TEM grids, the gel-like OM tends to spread along with the grids resulting in a thinness of these particles. This phenomenon can be clearly identified by the AFM analysis, as shown in Figs. S1b and c. The gel-like OM was thinner than the irregular OM which leads to the transparent morphology in TEM image (Fig. S1a).

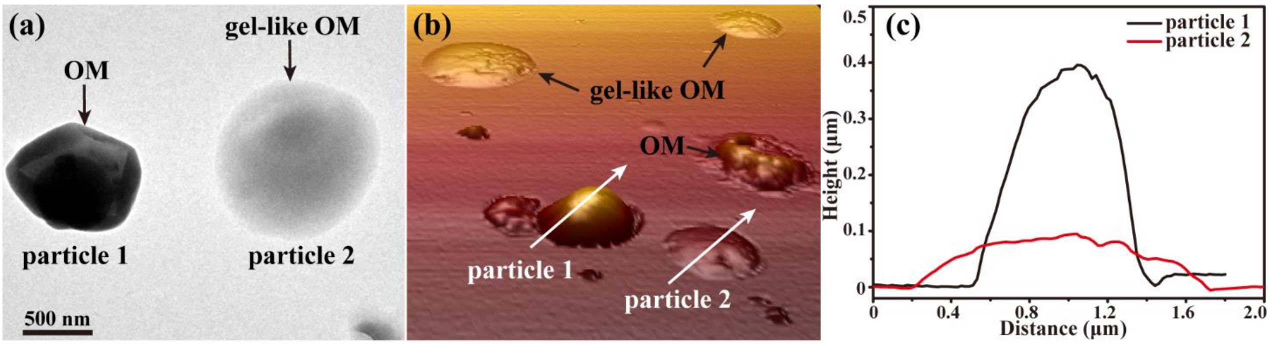

Figure S1. Morphology of irregular OM and gel-like OM obtained from TEM and AFM images. (a) TEM image of irregular OM (particle 1) and gel-like OM (particle 2); (b) AFM image of irregular OM and gel-like OM with particle 1 and 2 corresponding to that in (a) TEM image; (c) Height profiles of particle 1 and 2 corresponding to the line scan locations depicted by white lines in (b) AFM image.

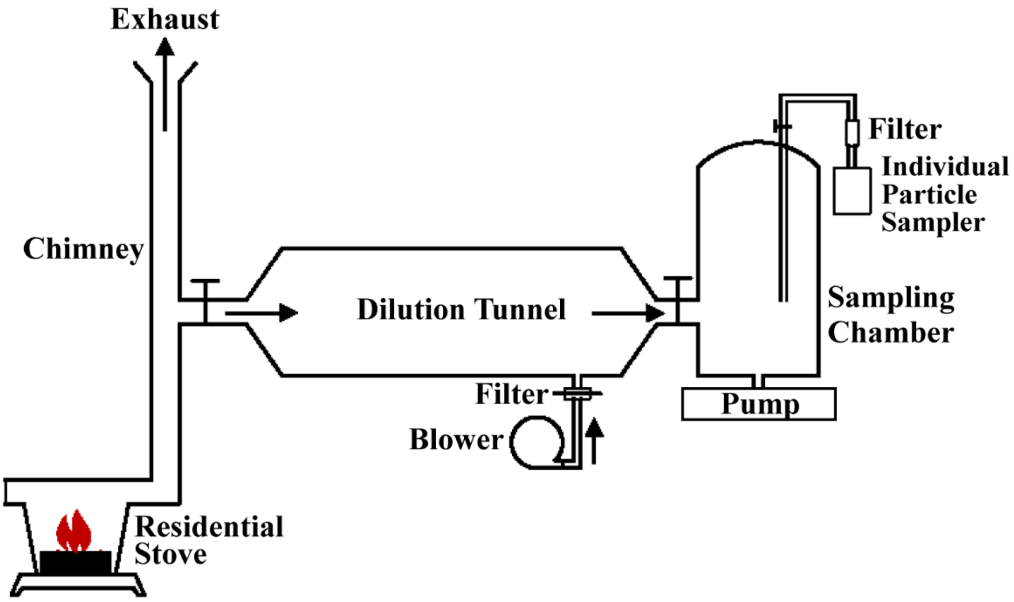

Figure S2. Schematic diagram of combustion facility.

Reference

1. Adachi, K. & Buseck, P. R. Atmospheric tar balls from biomass burning in Mexico. *Journal of Geophysical Research: Atmospheres* **116**, D05204, doi:10.1029/2010JD015102 (2011).
